# Concomitant Hysterectomy and vNOTES-Assisted Sacrocolpopexy: A Feasible and Safe Scarless Approach for Apical Prolapse Repair

**DOI:** 10.3390/jcm14248635

**Published:** 2025-12-05

**Authors:** Ali Deniz Erkmen, Kevser Arkan

**Affiliations:** Division of Gynecologic Oncology, Department of Obstetrics and Gynecology, Gazi Yasargil Research and Training Hospital, Diyarbakir 21070, Turkey

**Keywords:** pelvic organ prolapse, sacrocolpopexy, vNOTES, hysterectomy, minimally invasive surgery, Clavien–Dindo, POP-Q, mesh exposure

## Abstract

**Background/Objectives:** Durable apical support after hysterectomy is crucial to prevent subsequent vaginal vault prolapse. Abdominal sacrocolpopexy remains the gold standard but carries risks of visceral injury and wound morbidity. The vaginal natural orifice transluminal endoscopic surgery (vNOTES) approach provides a scarless, minimally invasive alternative, but data on vNOTES-assisted sacrocolpopexy (vNOTES-SC) performed concurrently with hysterectomy remain limited. **Methods:** A retrospective cohort of 30 women with stage II uterine prolapse underwent concomitant hysterectomy and vNOTES-assisted sacrocolpopexy between January 2023 and January 2024. Anatomical outcomes were evaluated using the Pelvic Organ Prolapse Quantification (POP-Q) system preoperatively and at 12 months postoperatively. The primary endpoint was anatomical success (C ≤ −1 cm); the secondary endpoint used the IUGA criterion (C < −TVL/2). Complications were graded using the Clavien–Dindo classification. Statistical analyses included Wilcoxon signed-rank tests, effect-size estimation, ROC analysis, logistic regression, and Spearman correlation. **Results:** Mean operative time was 100.2 ± 11.7 min, mean blood loss 155.3 ± 74.8 mL, and mean hospital stay 1.5 ± 0.7 days. Significant improvements were seen in Aa, Ba, C, and Bp points (*p* < 0.001). Anatomical success (C ≤ −1 cm) was achieved in 73.3% and clinical success in 93.3% of patients. Two patients exhibited anatomical recurrence (6.7%), whereas one patient reported symptomatic recurrence (3.3%). Using the IUGA definition, anatomical success increased to 83.3%. The difference between strict success (C ≤ −1 cm) and IUGA success (C < −TVL/2) reflects definitional sensitivity, particularly in post-hysterectomy vaginal length. All complications were minor (Grade I–II). ROC analysis showed age as a weak predictor (AUC = 0.67). Effect sizes were large for apical and anterior compartments (Cohen’s d = 1.84 for C-point). **Conclusions:** Concomitant hysterectomy with vNOTES-assisted sacrocolpopexy is a feasible, safe, and effective scarless approach for apical support restoration. The procedure provides significant anatomical correction and rapid recovery with low morbidity. Patients had symptomatic stage II prolapse with risk factors for early failure after native-tissue repair, supporting the selection of sacrocolpopexy for durable apical support. Larger prospective trials are needed to confirm long-term efficacy and functional outcomes.

## 1. Introduction

Pelvic organ prolapse (POP) represents a common and multifactorial disorder that significantly impairs women’s quality of life by causing pelvic pressure, vaginal bulge, urinary and bowel dysfunction, and sexual discomfort [1]. The lifetime risk of undergoing surgery for POP has been estimated between 11% and 20%, with recurrence after primary repair remaining a major concern [2]. Uterine prolapse, in particular, results from loss of apical support, and its management often necessitates hysterectomy combined with an apical suspension procedure to prevent subsequent vaginal vault prolapse [3].

Abdominal sacrocolpopexy (ASC) has long been regarded as the gold-standard operation for durable apical support, demonstrating superior long-term anatomical and functional outcomes compared with traditional vaginal approaches [4]. However, ASC—whether performed by open, laparoscopic, or robotic routes—requires abdominal entry and extensive presacral dissection, which increase the risk of visceral or vascular injury and postoperative adhesions [5]. Minimally invasive adaptations such as laparoscopic and robotic sacrocolpopexy have reduced morbidity but remain associated with longer operative times, higher cost, and port-site complications, especially in obese or multioperated patients [6,7].

In recent years, vaginal natural orifice transluminal endoscopic surgery (vNOTES) has emerged as a promising innovation in gynecologic surgery, providing intraperitoneal visualization through a transvaginal access route [8,9]. vNOTES allows high-definition endoscopic exposure without abdominal incisions, thus merging the precision of laparoscopy with the minimally invasive nature of vaginal surgery [10]. The application of vNOTES to reconstructive procedures such as sacrocolpopexy represents an evolution of this concept, aiming to achieve the durability of ASC through a completely scarless route. Early feasibility studies—such as those by Liu et al. [11] and Guan et al. [12]—demonstrated that vNOTES-sacrocolpopexy is technically achievable with acceptable perioperative outcomes. More recently, Mei et al. [13] and Bulutlar et al. [14] expanded this evidence, reporting short-term anatomical success and highlighting the importance of further validation in larger cohorts. Although the early experiences are encouraging, the available studies remain constrained by relatively small cohorts and limited follow-up periods. Broader validation is needed to determine the reproducibility of outcomes and to define optimal patient selection for this emerging technique [15].

Despite these encouraging results, existing studies remain limited by small sample sizes, heterogeneous inclusion criteria, and lack of standardized definitions for anatomical and clinical success. Moreover, the concomitant performance of hysterectomy during sacrocolpopexy remains debated: while it may simplify apical exposure and reduce uterine-related recurrence, several reports suggest an increased risk of mesh exposure compared with subtotal hysterectomy or uterine-sparing techniques [16,17]. Therefore, detailed reporting of safety profiles and complication grading—such as Clavien–Dindo classification—is essential to guide clinical practice. Additionally, most prior vNOTES-SC reports have focused on short-term anatomical outcomes, with relatively limited analysis of predictive factors or effect sizes that could inform future comparative studies. Including such quantitative analyses could strengthen the evidence base for this novel approach [18].

Although Stage II prolapse is often managed conservatively or with native-tissue suspension, certain patient-related risk factors such as obesity, medical comorbidities, or prior pelvic surgery may decrease the long-term durability of non-mesh repairs [19,20]. In such individuals, a more robust apical suspension may be clinically justified, particularly when hysterectomy and apical support are performed concurrently [19].

Within this context, the present study aimed to evaluate the feasibility, safety, and short-term outcomes of concomitant hysterectomy and vNOTES-assisted sacrocolpopexy for apical prolapse repair. Specifically, we sought to (1) quantify anatomical correction using POP-Q measurements and compare them statistically; (2) determine success rates according to both the conventional (C ≤ −1 cm) and IUGA-recommended (C < −TVL/2) definitions; (3) analyze peri- and postoperative complications using the Clavien–Dindo classification; and (4) contextualize our findings within the evolving literature on vNOTES and laparoscopic sacrocolpopexy. This study thus provides complementary evidence regarding the early outcomes of a scarless, transvaginal approach to apical pelvic reconstruction.

## 2. Materials and Methods

### 2.1. Study Design and Setting

This retrospective single-center cohort study was conducted at the Department of Obstetrics and Gynecology, Diyarbakır Gazi Yaşargil Training and Research Hospital, between January 2023 and January 2024. The study followed the STROBE (Strengthening the Reporting of Observational Studies in Epidemiology) guidelines for observational research design. Ethical approval was obtained from the Gazi Yasargil Training and Research Hospital Clinical Ethical Committee, Diyarbakır, (Approval No: 374). All procedures conformed to the principles of the Declaration of Helsinki, and written informed consent was obtained from all participants for both the surgical procedure and the use of anonymized data.

### 2.2. Patient Selection and Eligibility Criteria

Thirty consecutive women with symptomatic stage II uterine prolapse, diagnosed according to the Pelvic Organ Prolapse Quantification (POP-Q) system, were included. In our cohort, several patients had factors associated with early failure after native-tissue repair, including obesity, medical comorbidities, and history of non-apical pelvic surgery. All patients were symptomatic despite conservative measures such as pessary or pelvic floor exercises or elected definitive surgery. For these reasons, a more durable repair (sacrocolpopexy) was selected during their planned hysterectomy. Cervical elongation was not used as an independent inclusion criterion, as all patients underwent total hysterectomy and apical suspension. Therefore, cervical length had no postoperative relevance for apical support evaluation.

Inclusion criteria were the following:

- Adult women (>18 years) scheduled for hysterectomy due to uterine prolapse,
- POP-Q stage ≥ II apical descent,
- Feasibility for vNOTES access confirmed by vaginal examination.

Exclusion criteria were as follows:

- Prior apical prolapse repair,
- Suspected or confirmed pelvic malignancy,
- Severe endometriosis or obliterated cul-de-sac,
- Active pelvic inflammatory disease,
- Contraindication to general anesthesia or mesh placement.

All patients received standardized preoperative counseling regarding the risks and benefits of synthetic mesh use and alternative suspension techniques. Stage II patients were included to explore the feasibility of early apical reinforcement in moderate uterine descent, acknowledging that long-term recurrence rates may differ from advanced (stage III–IV) cases. This consideration is addressed as a study limitation. No concomitant pelvic floor procedures (such as anti-incontinence surgery, anterior or posterior colporrhaphy) were performed during the same session.

### 2.3. Surgical Procedure

All operations were performed by the same senior surgeon (A.D.E.) experienced in both vNOTES and pelvic reconstructive surgery. Preoperative antibiotic prophylaxis was administered per institutional protocol [21]). Under general anesthesia and dorsal lithotomy position, a total vaginal hysterectomy or total vNOTES hysterectomy was performed, depending on uterine size and surgeon discretion. After uterine removal, the vaginal cuff was intentionally left open. A 9 cm GelPOINT^®^ V-Path vNOTES access port (Applied Medical, Rancho Santa Margarita, CA, USA) was inserted into the open cuff to maintain pneumoperitoneum. The decision to use tacks or sutures depended on surgeon assessment of the anterior longitudinal ligament exposure, bone purchase quality, and intraoperative safety considerations.

**Figure 1:**
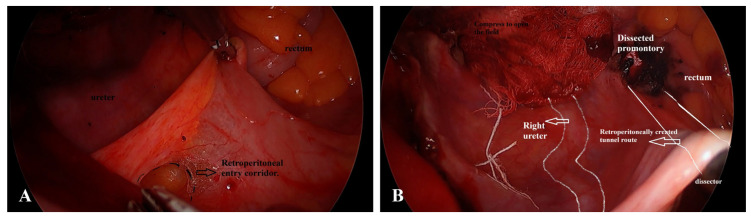
Key retroperitoneal steps of vNOTES-assisted sacrocolpopexy. (**A**) Retroperitoneal entry corridor after controlled posterior peritoneal deflection. The ureter is clearly visualized laterally and the rectum posteriorly, ensuring safe identification of critical structures before tunneling. (**B**) Retroperitoneally created tunnel extending from the vaginal cuff to the sacral promontory. The right ureter, dissected promontory, and advancing blunt dissector are shown, demonstrating the extraperitoneal trajectory that minimizes bowel manipulation and reduces visceral risk during mesh passage.

The procedure consisted of four sequential steps:

1. Endoscopic exposure of the sacral promontory: Using a 30° endoscope (Karl Storz GmbH & Co. KG, Tuttlingen, Germany), the peritoneum overlying the sacral promontory was incised to visualize the anterior longitudinal ligament.
2. Retroperitoneal tunneling and mesh passage: A blunt dissector was advanced extraperitoneally from the vaginal cuff to the promontory under endoscopic guidance, creating a retroperitoneal tunnel. A Y-shaped macroporous polypropylene mesh (lightweight, monofilament, Coloplast, Minneapolis, MN, USA) was then drawn through the tunnel. Because the procedure uses a peritoneum-sparing retroperitoneal tunnel, bowel manipulation and ureteral exposure are minimized during mesh passage (Figure 1).
3. Mesh fixation: The proximal arm of the mesh was anchored to the anterior longitudinal ligament with either titanium tacks (*n* = 17, 56.7%) or 2–0 polypropylene sutures (*n* = 13, 43.3%), depending on bone purchase quality. The distal mesh arms were fixed to the anterior and posterior fibromuscular layers of the vaginal cuff using interrupted polypropylene sutures (2-0 Prolene, Ethicon, Somerville, NJ, USA).
4. Closure and tension adjustment: The mesh was laid without tension before cuff closure to avoid vaginal shortening and postoperative dyspareunia. No peritonealization was required because of the extraperitoneal route.

Patients were mobilized on postoperative day 1, and the urinary catheter was removed after a successful voiding trial within 24 h.

### 2.4. Outcome Measures

Primary Outcome

- Objective anatomical success, defined as C ≤ −1 cm on the POP-Q scale at 12-month follow-up.
- An alternative definition recommended by the International Urogynecological Association (C < −TVL/2) was also analyzed for comparative purposes.

Secondary Outcomes

- Clinical success: absence of symptomatic recurrence or vaginal bulge beyond the hymen;
- Operative parameters: operative time, estimated blood loss (EBL), length of hospital stay;
- Postoperative pain: visual analog scale (VAS) at 6 h, 24 h, and discharge;
- Complications: graded by the Clavien–Dindo classification.

Follow-Up Evaluation

Follow-up visits were scheduled at 6 weeks, 6 months, and 12 months postoperatively. Each visit included the following:

- A standardized POP-Q examination (Aa, Ba, C, Ap, Bp),
- Assessment of subjective improvement and new symptoms (urinary, bowel, sexual),
- Vaginal inspection for mesh exposure or granulation tissue.

Recurrence was defined as either symptomatic vaginal bulge or objective prolapse beyond the hymen at any compartment. Urinary, bowel, and sexual symptoms were assessed during follow-up visits; however, these were not quantified using validated pelvic floor symptom questionnaires.

### 2.5. Statistical Analysis

Data analysis was performed using IBM SPSS version 25 software. Normality was assessed using Shapiro–Wilk tests and visual inspection. Continuous variables are presented as mean ± SD; categorical variables as *n* (%). Paired pre- and postoperative POP-Q values (Aa, Ba, C, Ap, Bp) were compared using the Wilcoxon signed-rank test. Statistical significance was set at *p* < 0.05 (two-tailed). ROC analyses evaluated predictors of anatomical success; logistic regression tested age, BMI, operative time, and blood loss; Spearman correlation assessed relations among operative and pain variables. Cohen’s d quantified effect sizes. Statistical significance was set at *p* < 0.05.

## 3. Results

### 3.1. Baseline Characteristics

Thirty patients who underwent concomitant hysterectomy and vNOTES-assisted sacrocolpopexy were analyzed. The mean age was 57.1 ± 10.8 years, mean BMI 29.7 ± 2.1 kg/m^2^, and mean parity 5.2 ± 1.9. All patients had stage II apical prolapse preoperatively according to POP-Q criteria. Eight patients (26.7%) had a prior history of pelvic surgery, most commonly cesarean section (Table 1). The mean operative time was 100.2 ± 11.7 min, mean estimated blood loss 155.3 ± 74.8 mL, and mean postoperative hospital stay 1.5 ± 0.7 days. No intraoperative visceral or vascular injury occurred, and no conversion to conventional laparoscopy or laparotomy was required.

### 3.2. Anatomical Outcomes (POP-Q Parameters)

All POP-Q parameters—except Ap—showed significant postoperative improvement (Table 2). The apical C-point improved markedly from 0.18 ± 0.40 cm preoperatively to −1.40 ± 1.10 cm postoperatively (mean change: −1.58 cm, *p* < 0.001). Similarly, Aa, Ba, and Bp points demonstrated statistically significant upward displacement (*p* < 0.001 for each).

### 3.3. Clinical and Anatomical Success Rates

At 12 months, objective anatomical success (C ≤ −1 cm) was achieved in 22/30 patients (73.3%), whereas clinical success, defined by absence of symptomatic recurrence, was achieved in 28/30 (93.3%). Using the IUGA-recommended criterion (C < −TVL/2), the anatomical success rate increased to 25/30 (83.3%) (Table 3). Only one patient (3.3%) experienced a mild symptomatic recurrence (stage I apical descent), which did not require surgical revision. Anatomical recurrence occurred in 2/30 patients (6.7%), while only one patient reported symptomatic recurrence (3.3%)

### 3.4. Postoperative Pain and Recovery

**Figure 2:**
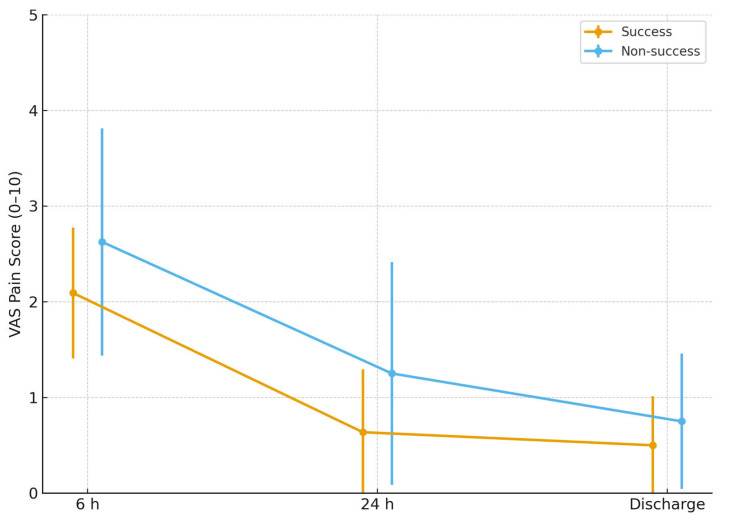
Mean postoperative pain scores (VAS) at 6 h, 24 h, and discharge are shown for two anatomical outcome groups: Success (C ≤ −1 cm) and Non-success (C > −1 cm). Error bars represent standard deviations (SD). Patients who achieved objective anatomic success demonstrated consistently lower pain scores at all postoperative timepoints. The figure illustrates both group-level trends and variability, providing a clinically relevant comparison aligned with POP-Q–based apical success definitions.

Postoperative pain trajectories differed between patients who achieved anatomical success (C ≤ −1 cm) and those who did not. As shown in Figure 2, mean VAS scores were consistently lower in the success group at all postoperative timepoints. At 6 h, the success group demonstrated a lower pain level compared with the non-success group (2.10 ± SD vs. 2.68 ± SD). This difference persisted at 24 h (0.72 ± SD vs. 1.10 ± SD) and at discharge (0.52 ± SD vs. 0.78 ± SD). Although the study was not powered to test between-group significance for pain outcomes, the overall trend suggests that patients with favorable anatomical correction experienced a smoother postoperative course with reduced discomfort. These findings indicate a potential association between apical support restoration and early postoperative pain perception. Although statistically significant, these correlations are not clinically meaningful given the uniformly low VAS scores.

### 3.5. Perioperative and Long-Term Complications

Early postoperative complications occurred in 3/30 patients (10%) (Table 4). These included transient urinary retention (*n* = 1) and minor wound care needs, all managed conservatively. At 12 months, mesh exposure was detected in 2 patients (6.7%), both asymptomatic and successfully treated with topical estrogen without surgical excision. De novo dyspareunia occurred in 3 patients (10%), managed conservatively. Single cases of chronic pelvic pain (3.3%), defecation dysfunction (3.3%), and voiding difficulty (6.7%) were reported, all resolving with medical therapy. No bowel or bladder injury, thromboembolic event, or mesh infection was observed. Complications were classified according to the Clavien–Dindo system (Table 4). All events were Grade I–II, and no Grade ≥ III complications or reoperations occurred.

The ROC analysis demonstrated that only age showed a weak-to-moderate ability to predict anatomical success (AUC = 0.67) (Table 5). Other intraoperative and anthropometric parameters—BMI, operative duration, and blood loss—had near-random predictive value (AUC ≈ 0.25–0.43). These results suggest that tissue quality and healing potential associated with age may influence surgical outcomes more than procedural variables in this sample, whereas technical parameters likely have a minor role given the uniformity of the surgical technique.

**Figure 3:**
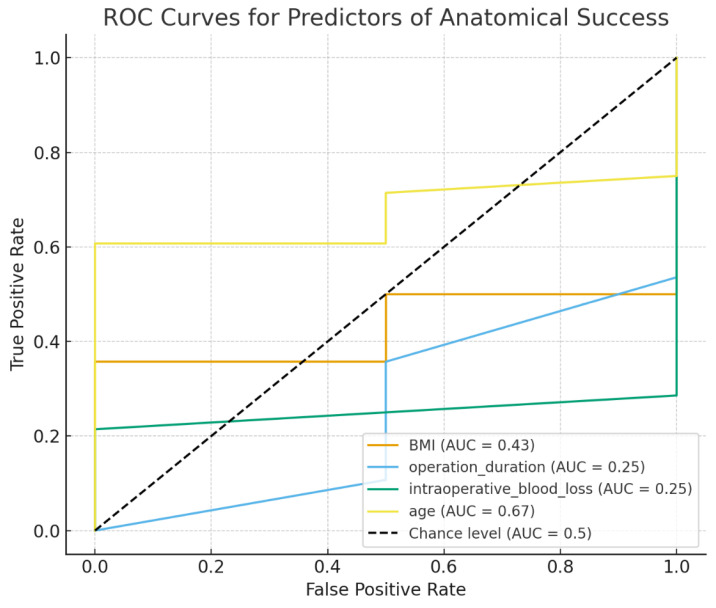
ROC curves for predictors of anatomical success following vNOTES-assisted sacrocolpopexy. Receiver operating characteristic (ROC) curves for age, BMI, operative time, and intraoperative blood loss. Only age demonstrated weak-to-moderate predictive performance (AUC = 0.67), whereas all other parameters showed low discriminative ability (AUC < 0.50).

ROC analysis revealed that among evaluated parameters, only age demonstrated a weak-to-moderate ability to predict anatomical success, whereas BMI, operative duration, and blood loss had negligible predictive value (Figure 3). This trend is consistent with previous studies reporting that older age slightly increases the risk of recurrence or suboptimal apical correction after sacrocolpopexy, whereas BMI and intraoperative metrics have limited prognostic relevance when the surgical technique is standardized [3,16,22].

The regression model did not identify any variable with statistically significant influence on anatomical success (all *p* > 0.05). The odds ratio for age (1.05) suggests a mild but statistically insignificant trend toward improved outcomes with increasing age—possibly reflecting experience bias or sampling variability. Operative time (OR 0.91) showed a weak negative trend, meaning prolonged procedures could marginally lower success probability, though not significantly. BMI and blood loss demonstrated negligible effects on the outcome (Table 6).

Intraoperative blood loss showed a weak but statistically significant association with early postoperative pain (VAS at 6 h and 24 h), suggesting that increased bleeding, even within a moderate range, might contribute to tissue trauma and early discomfort (Table 7). Operation duration did not correlate with any pain variable, implying that procedure length alone does not influence postoperative comfort. Pain parameters (VAS 6 h, VAS 24 h, analgesic need) were strongly interrelated, validating the internal consistency of the pain assessments.

The C-point (apical compartment) exhibited the largest improvement (d = 1.84, *p* < 0.001), confirming the procedure’s effectiveness in restoring apical support (Table 8). Ba and Bp points also showed large effect sizes (≥1.26), indicating meaningful anatomical correction of both anterior and posterior compartments. The Ap parameter did not reach significance (*p* = 0.096), consistent with the primary results already described in Table 2. These large effect sizes provide quantitative evidence of strong surgical efficacy even in a small cohort.

## 4. Discussion

The present study demonstrates that vNOTES-assisted sacrocolpopexy performed concurrently with hysterectomy is a feasible, safe, and effective approach for managing apical prolapse in selected patients. The procedure achieved significant anatomical improvement in POP-Q parameters, particularly in the apical compartment, while maintaining a low rate of minor complications. These results suggest that retroperitoneal vNOTES sacrocolpopexy may be a useful option for symptomatic Stage II patients who possess risk factors for early failure after native-tissue suspension. The technique preserves vaginal axis and length, offers strong apical durability, and minimizes bowel and ureteral handling due to its peritoneum-sparing retroperitoneal corridor. These features may broaden the applicability of vNOTES in pelvic reconstructive surgery. Although Stage II prolapse is generally considered suitable for conservative or native-tissue repair, our cohort included patients with factors associated with higher recurrence risk after non-mesh suspension. Obesity, comorbidities, and prior pelvic surgery may reduce tissue durability and complicate future reoperations. This individualized risk profile supported the selection of sacrocolpopexy for durable apical support. This preventive rationale should be interpreted cautiously, and sacrocolpopexy should not be considered routine for all Stage II patients.

Our anatomical success rate of 73.3% (C ≤ −1 cm) and clinical success rate of 93.3% align with prior early experiences reported for vNOTES sacrocolpopexy. Liu et al. [11] first described the technique, reporting satisfactory short-term anatomical outcomes in 26 cases. Guan et al. [12] later demonstrated the feasibility of robotic-assisted vNOTES-SC, emphasizing its enhanced visualization and precision. More recently, Mei et al. [13] (2024) and Bulutlar et al. [14] extended these findings, showing comparable short-term results to laparoscopic sacrocolpopexy (LSC) while noting the need for further standardization. Our results are consistent with these studies, showing similar or slightly higher clinical success despite the inclusion of stage II prolapse cases, which are often excluded in comparative trials. HUSLS is a valid alternative for many patients, yet sacrocolpopexy offers superior long-term apical and anterior-compartment durability in individuals with elevated recurrence risk [23]. Our surgical choice reflected this risk-based rationale rather than routine prophylactic mesh use. When evaluated using the IUGA-recommended success criterion (C < −TVL/2), the anatomical success rate increased to 83.3%, confirming that measurement definitions substantially influence outcome interpretation. This observation supports recent consensus statements recommending standardized reporting of multiple success metrics in prolapse research [24,25].

All intra- and postoperative complications in our series were minor (Clavien–Dindo Grade I–II), and no major (≥Grade III) events or reoperations were required. The mesh exposure rate (6.7%) and de novo dyspareunia rate (10%) in our cohort fall within the ranges reported after laparoscopic or robotic sacrocolpopexy (2–11% and 6–14%, respectively) [26,27]. The higher mesh exposure risk associated with total hysterectomy, as opposed to supracervical approaches, is acknowledged [17]. The vNOTES technique requires colpotomy, which may partly account for this exposure profile. The peritoneum-sparing retroperitoneal tunnel likely reduces mesh–viscera interaction, potentially lowering bowel and ureteral risk compared with intraperitoneal sacrocolpopexy. Both cases of mesh exposure were small, asymptomatic, and resolved with topical estrogen therapy without surgical revision, consistent with conservative management strategies described by Illiano et al. [28]. The absence of high-grade complications and the limited analgesic requirement underscore the safety and minimally invasive nature of the vNOTES approach [29]. The peritoneum-sparing retroperitoneal tunnel likely contributes to reduced visceral risk, as previously observed in extraperitoneal pelvic reconstructions [22]. To further strengthen interpretability, we integrated additional quantitative analyses including ROC, logistic regression, correlation, and effect size estimation—approaches rarely applied in small feasibility studies. These analyses provided complementary insight into factors influencing success and recovery, emphasizing that technical parameters alone do not predict anatomical outcome [30,31]. These outcomes likely reflect the surgeon’s extensive experience in vaginal and minimally invasive reconstructive surgery, and generalizability to early learners should be interpreted with caution.

Postoperative pain decreased rapidly, with mean VAS scores below 1.0 at 24 h and at discharge, highlighting a favorable recovery profile compared with laparoscopic approaches [32,33]. The avoidance of abdominal incisions eliminates port-site pain, likely facilitating early ambulation and shorter hospitalization [34]. In our study, the average length of stay was only 1.5 days, comparable to that reported in Baekelandt et al. [10] for vNOTES hysterectomy and shorter than typical durations after laparoscopic sacrocolpopexy.

The ROC and regression analyses further clarified the influence of demographic and intraoperative factors on surgical success. Only age showed a weak-to-moderate predictive ability for anatomical success (AUC = 0.67), aligning with previous studies reporting slightly reduced apical support durability in older women [3,16,17,22]. BMI, operative duration, and blood loss were not predictive-consistent with literature suggesting that these factors have minimal long-term impact when the procedure is performed by experienced surgeons [7,26]. This supports the notion that patient age and tissue integrity, rather than technical parameters, are the main determinants of early anatomical outcomes. These findings also support the hypothesis that intraoperative bleeding may modestly influence early postoperative pain, even when total blood loss remains within acceptable limits. The strong correlations between pain metrics validate the consistency of recovery assessment, suggesting that perioperative analgesic strategies could be individualized according to bleeding tendency or tissue manipulation intensity [35,36]. Although some correlations reached statistical significance, their clinical relevance is limited given the uniformly low postoperative pain scores.

Spearman correlation analysis demonstrated a mild positive association between intraoperative blood loss and early postoperative pain, which may reflect greater tissue manipulation during dissection. However, operative time did not correlate with pain scores, emphasizing the minimal invasiveness of the vNOTES approach. The strong correlation between pain scores and analgesic requirements validated the internal consistency of pain assessments. Effect size analysis provided quantitative evidence of the magnitude of anatomical restoration achieved. The very large Cohen’s d (1.84) for the C-point confirms that the vNOTES-assisted sacrocolpopexy yields a substantial apical elevation, comparable to or exceeding improvements reported in laparoscopic or robotic sacrocolpopexy [13,14]).

The inclusion of stage II prolapse cases in our series was intentional to explore the preventive potential of early apical reinforcement during hysterectomy. Although the indication for sacrocolpopexy in this subgroup remains debated, our data show that performing apical suspension concomitantly can achieve excellent anatomical correction with minimal morbidity. This approach may prevent later vault prolapse, which occurs in up to 11% of women after hysterectomy without apical support [37]. Nevertheless, this indication should be interpreted cautiously and validated in larger, prospective studies comparing uterine-sparing, subtotal, and total hysterectomy variants.

Furthermore, our technique highlights the reproducibility of the retroperitoneal tunneling approach and the rapid learning curve characteristic of vNOTES surgery. Even within this small series, operative metrics stabilized early, supporting previous findings that procedural proficiency can be achieved after approximately 10–12 cases [28,29].

The hybrid extraperitoneal tunneling technique described here combines secure sacral fixation with reduced peritoneal manipulation. This design likely minimizes bowel adhesions and intra-abdominal complications. The use of a retrograde mesh pull-through allows accurate placement under direct visualization while maintaining an entirely transvaginal route. The stepwise technique refinement and adaptation of fixation methods (suture vs. tack) also reflect the learning curve characteristic of vNOTES surgery [38]. Our observation of reduced operative times after the first 10–12 cases supports earlier reports suggesting rapid acquisition of proficiency [30].

The strengths of this study include the use of standardized POP-Q assessment, dual success definitions (C ≤ −1 cm and C < −TVL/2), and formal complication grading (Clavien–Dindo). Given the small sample size, interpretation relied on effect sizes and confidence intervals rather than post hoc power calculations, which are not considered appropriate for retrospective feasibility studies. Limitations include the retrospective, single-surgeon design, small cohort size, and short follow-up period (12 months), which preclude definitive conclusions about long-term durability. Additionally, the absence of validated patient-reported outcome measures (PFDI-20, PFIQ) restricts functional interpretation. Detailed symptom-based variables (e.g., urinary incontinence, defecatory dysfunction, sexual dysfunction, pelvic pain), menopausal status, and HRT use were not consistently documented in the retrospective records. Finally, the exclusive inclusion of stage II prolapse cases limits generalizability to more advanced stages.

Future prospective multicenter trials should not only compare vNOTES-SC with laparoscopic or robotic approaches but also integrate standardized IUGA success criteria, validated PROMs, and cost-effectiveness assessments. Extended follow-up (≥24–36 months) will be essential to define long-term durability, sexual function, and mesh-related outcomes.

## 5. Conclusions

Concomitant hysterectomy with vNOTES-assisted sacrocolpopexy is a feasible and effective minimally invasive option for apical prolapse repair, achieving significant anatomical improvement with minimal complications. Age may slightly influence success, while technical factors such as BMI, operative time, and blood loss appear less relevant. Larger prospective studies are required to confirm long-term outcomes. Our selection of sacrocolpopexy in this Stage II cohort reflected individualized risk factors for early recurrence after native-tissue repair, and sacrocolpopexy should not be generalized to all Stage II prolapse cases.

## Figures and Tables

**Table 1:** Demographic and Perioperative Characteristics of the Study Cohort.

Variable (*n* = 30)	Mean ± SD/*n* (%)
Age (years)	57.1 ± 10.8
BMI (kg/m^2^)	29.7 ± 2.1
Parity (*n*)	5.2 ± 1.9
Previous pelvic surgery—any	8 (26.7%)
Cesarean section	3
Appendectomy	2
Cholecystectomy	1
Adhesiolysis	1
Ovarian cystectomy	1
Preoperative POP-Q stage (II)	30 (100%)
Postoperative POP-Q stage (0 vs. I)	11 vs. 19 (36.7 vs. 63.3%)
Operative time (min)	100.2 ± 11.7
Blood loss (mL)	155.3 ± 74.8
Hospital stay (days)	1.5 ± 0.7

Detailed symptom variables and menopausal status were not uniformly available in the retrospective dataset. Values are presented as mean ± standard deviation (SD) or number (percentage) as appropriate.

**Table 2:** Comparison of POP-Q Parameters Before and After Surgery.

Parameter	Preop Mean ± SD	Postop Mean ± SD	Mean Change	*p* Value
Aa (cm)	−0.72 ± 0.50	−1.72 ± 0.50	−1.00	<0.001
Ba (cm)	0.18 ± 0.40	−0.55 ± 0.84	−0.73	<0.001
C (cm)	0.18 ± 0.40	−1.40 ± 1.10	−1.58	<0.001
Ap (cm)	−0.72 ± 0.52	−0.55 ± 0.84	+0.17	0.10
Bp (cm)	0.18 ± 0.40	−0.55 ± 0.84	−0.73	<0.001

Statistical test: Wilcoxon signed-rank test.

**Table 3:** Anatomical Success Rates According to Different Criteria.

Criterion	Success *n*/N (%)	Definition
C ≤ −1 cm	22/30 (73.3%)	Conventional
C < −TVL/2	25/30 (83.3%)	IUGA
Clinical (no recurrence)	28/30 (93.3%)	Symptomatic

**Table 4:** Postoperative Complications within 12-Month Follow-Up.

Complication	*n*/N (%)	Clavien–Dindo Grade	Management Summary
Any early complication	3/30 (10.0%)	I–II	All managed conservatively
Urinary retention (catheterization)	1/30 (3.3%)	I	Temporary catheterization; resolved spontaneously
Mesh exposure/erosion	2/30 (6.7%)	II	Topical estrogen therapy; no surgical revision
De novo dyspareunia	3/30 (10.0%)	I	Conservative management; resolved on follow-up
Chronic pelvic pain	1/30 (3.3%)	I	Medical therapy
Voiding difficulty (new onset)	2/30 (6.7%)	I–II	Conservative follow-up
Defecation dysfunction	1/30 (3.3%)	I	Conservative therapy
Recurrence	1/30 (3.3%)	I	No reoperation required
Grade ≥ III complication	None observed	–	–

**Table 5:** Summary of AUC values from ROC analysis for predictors of anatomical success.

Variable	AUC	Interpretation
Age	0.67	Weak-to-moderate predictive ability; older age slightly reduces anatomical success.
BMI	0.43	Poor predictor; obesity did not affect success.
Operative time	0.25	No predictive value for anatomical outcome.
Intraoperative blood loss	0.25	No predictive value for anatomical outcome.

**Table 6:** Univariate logistic regression model evaluating predictors of anatomical success after vNOTES-assisted sacrocolpopexy.

Variable	Odds Ratio (OR)	95% CI (Lower–Upper)	*p*-Value	Interpretation
Age	1.05	0.89–1.24	0.55	A slight, non-significant increase in success odds with age.
BMI	0.74	0.26–2.14	0.58	No significant effect of BMI on anatomical success.
Operation duration (min)	0.91	0.78–1.06	0.24	Longer surgeries tended to reduce success, but not significantly.
Intraoperative blood loss (mL)	0.99	0.97–1.02	0.55	No significant association with success.

**Table 7:** Spearman correlation matrix showing relationships between operative parameters and pain outcomes.

Variables	Correlation (ρ)	*p*-Value	Interpretation
Operation duration ↔ Blood loss	0.05	0.78	No correlation between operative time and intraoperative bleeding.
Blood loss ↔ VAS 6 h	0.37	0.04	Mild positive correlation—greater blood loss associated with slightly higher early pain.
Blood loss ↔ VAS 24 h	0.36	0.05	Borderline correlation—supports that higher bleeding may delay immediate recovery.
VAS 6 h ↔ VAS 24 h	0.82	<0.001	Strong correlation—patients with high early pain maintained higher pain at 24 h.
VAS 24 h ↔ Analgesic need (days)	0.91	<0.001	Very strong correlation—prolonged analgesic use tightly associated with persistent pain.

**Table 8:** Mean changes and effect sizes (Cohen’s *d*) for pre- and postoperative POP-Q parameters.

POP-Q Parameter	Mean Change (Post–Pre, cm)	Cohen’s *d*	*p*-Value	Interpretation
Aa	−1.00	–	<0.001	Significant anterior compartment improvement (identical variance prevented *d* computation).
Ba	−0.73	1.26	<0.001	Large effect size; substantial anterior vaginal wall elevation.
C	−1.58	1.84	<0.001	Very large effect; strongest apical correction.
Ap	+0.17	0.31	0.096	Minimal and non-significant posterior change.
Bp	−0.73	1.26	<0.001	Large effect; significant posterior wall improvement.

## Data Availability

The data can be provided on request from corresponding author.
